# PLGA-Based Nanoparticles in Cancer Treatment

**DOI:** 10.3389/fphar.2018.01260

**Published:** 2018-11-02

**Authors:** Sima Rezvantalab, Natascha Ingrid Drude, Mostafa Keshavarz Moraveji, Nihan Güvener, Emily Kate Koons, Yang Shi, Twan Lammers, Fabian Kiessling

**Affiliations:** ^1^Department of Chemical Engineering, Amirkabir University of Technology (Tehran Polytechnic), Tehran, Iran; ^2^Institute for Experimental Molecular Imaging, University Clinic and Helmholtz Institute for Biomedical Engineering, RWTH Aachen University, Aachen, Germany; ^3^Department of Nuclear Medicine, Uniklinik RWTH Aachen University, Aachen, Germany; ^4^Department of Pharmacology and Toxicology, College of Pharmacy & UA Cancer Center, University of Arizona, Tucson, AZ, United States

**Keywords:** PLGA, nanoparticles, drug delivery, cancer treatment, combination therapy

## Abstract

Nanomedicines can be used for a variety of cancer therapies including tumor-targeted drug delivery, hyperthermia, and photodynamic therapy. Poly (lactic-co-glycolic acid) (PLGA)-based materials are frequently used in such setups. This review article gives an overview of the properties of previously reported PLGA nanoparticles (NPs), their behavior in biological systems, and their use for cancer therapy. Strategies are emphasized to target PLGA NPs to the tumor site passively and actively. Furthermore, combination therapies are introduced that enhance the accumulation of NPs and, thereby, their therapeutic efficacy. In this context, the huge number of reports on PLGA NPs used as drug delivery systems in cancer treatment highlight the potential of PLGA NPs as drug carriers for cancer therapeutics and encourage further translational research.

## Introduction

The field of nanomedicine, which refers to the application of nanotechnology in medicine, offers valuable tools for the diagnosis and treatment of diseases. In this regard, a wide range of submicron materials has been designed and engineered, especially for defeating cancer. Its applications expedite the development of contrast agents, therapeutics, drug delivery vehicles and theranostics. Nanoparticles (NPs) for drug delivery applications have been composed of biodegradable and biocompatible polymers based on natural and/or synthetic materials. Synthetic polymers can be produced with high purities in a precise and well-controlled production process, as compared to natural products (Lai et al., 2014).

One extensively investigated polymer is poly (lactic-co-glycolic acid) (PLGA), synthetic thermoplastic aliphatic biocompatible polyester. There are specific formulations based on PLGA and its related homopolymers; poly (lactic acid) (PLA) and poly (glycolic acid) (PGA), which have been approved by the US Food and Drug Administration (FDA) for medical applications (Pandey et al., 2015). PLGA NPs have also proved their potential as drug delivery systems for many therapeutic agents (e.g., chemotherapy, antibiotics, antiseptic, anti-inflammatory and antioxidant drugs, proteins), and can be favorable for tumor- and/or DNA-targeting (Danhier et al., 2012; Berthet et al., 2017).

**FIGURE 1:**
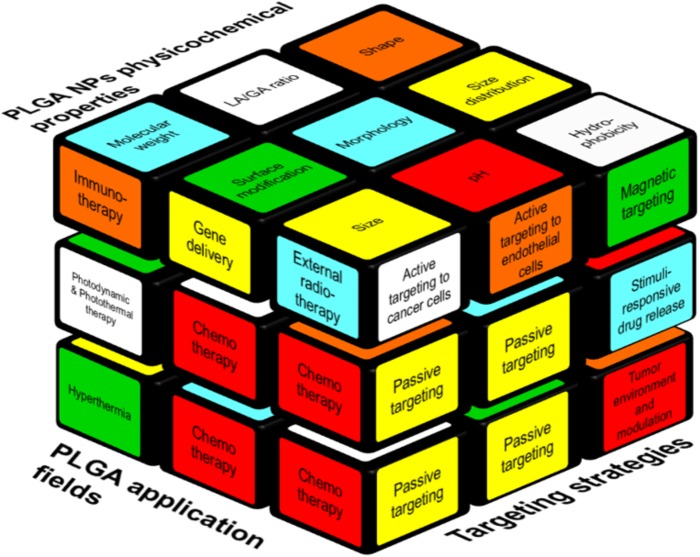
Interplay between various factors relevant to the design of PLGA-based NP formulations. The factors that are contributing to a successful cancer treatment by PLGA NPs are represented as a Rubik’s cube. Passive targeting is the cornerstone of the all targeting strategies (right side) beside other strategies like specific cancer cell- or endothelial cell targeting. Moreover, internal (e.g., pH) and external (e.g., heat, magnetic field) stimuli can be used for targeted delivery. Polymer and NPs properties such as size, molecular weight, hydrophobicity (upper side) play a role to formulate suitable carriers to deliver the drugs to the cancer site. On the left side, applications of PLGA NPs are reported, of which the use as a chemotherapeutic cargo is most common.

Scientists are trying to find new and different methods of NPs preparation and modification to gain tight control over PLGA degradation, drug release, and other characteristics. In this regard, researchers are evaluating various targeting strategies including, e.g., active targeting moieties to improve the retention of NPs at the target site. However, the correlation between PLGA NPs’ physicochemical properties, targeting strategies and treatment modalities on cancer therapy resemble a complex puzzle like a Rubik’s cube. As illustrated in Figure 1 it is not possible to move (and change) one brick without moving a whole plane. On the upper face of the cube, we illustrate polymers’ properties, and NPs’ features that correlate with, e.g., NPs size, which is determined by the molecular weight of the polymer (Mw), charge, coiling, and the type of surface modification. Moreover, any change in lactic acid (LA)/glycolic acid (GA) ratio varies the crystallinity and hydrophobicity of the formulation.

While a Rubik’s cube can be solved by defined algorithms, analog algorithms do not yet exist for the design of drug delivery systems due to its high complexity. Optimizing and altering chemical parameters will as well influence the other parts of the puzzle. Polymers’ properties and NPs features can affect the choice of the targeting strategy, and *vice versa*. For example, an external stimuli-based targeting strategy facilitates the tumor retention of NPs but it can exacerbate the size and might stimulate uptake by the mononuclear phagocytic system (MPS) (Kunjachan et al., 2014; Al-Jamal et al., 2016). Those changes in biodistribution and pharmacokinetics are the “hidden” faces of the cube, which can only be manipulated to a certain (rather low) extent via co-medication or interventions, e.g., sonoporation (Dasgupta et al., 2016; Dimcevski et al., 2016; Koczera et al., 2017) or *in vivo* modulation of oxidative stress via administration of buthionine sulfoximine (BSO), an inhibitor of the antioxidant glutathione (GSH). GSH neutralizes reactive oxygen species (ROS) and its depletion cannot only prevent premature degradation of a redox-sensitive nanoformulation but can also serve as a hypoxic cell sensitizer in combination with radiation and some chemotherapeutic drugs (Drude et al., 2018; Miran et al., 2018).

However, even a regular Rubik’s cube does not represent the full complexity: in drug targeting with nanomedicines some blocks are connetced and this connection might even increase or decrease until a very late stage of a given study to reach the optimal treatment outcome.

In this review article, we will address the displayed aspects of this cube for PLGA-based NPs and we will discuss how they can be tailored by synthesis methods as well as strategies for drug delivery with PLGA to improve cancer treatment.

## PLGA Properties

Poly (lactic-co-glycolic acid) is one of the best characterized biodegradable copolymers that decomposes to non-toxic products (H_2_O and CO_2_) that are eliminated from the body. Its polymeric NP degrades *in vivo* through hydrolysis of the ester bonds to its monomeric anions (lactate and glycolate). While D-Lactate is not further metabolized before excretion, L-lactate is converted into CO_2_, which is excreted through the lungs and it is converted to pyruvate, which enters the Krebs cycle. Glycolate on the other hand is either directly excreted through the renal system or it can be oxidized to glyoxylate, which is afterward further converted into glycine, serine, and pyruvate. The latter can again enter the Krebs cycle and is metabolized into CO_2_ and H_2_O. (Danhier et al., 2012; Silva et al., 2015). Typically, PLGA is produced by a catalyzed ring-opening copolymerization of LA and GA (Dechy-Cabaret et al., 2004). PGA is a crystalline hydrophilic polymer with low water solubility and fast degradation rate under physiological conditions. On the contrary, PLA is a stiff and hydrophobic polymer with low mechanical strength. As a copolymer of both, PLGA inherits the intrinsic properties of its constitutional monomers where the polymeric content, based on LA/GA ratio and Mw, strongly affect its degradation rate. For example, with an increase in the LA/GA ratio, the overall PLGA hydrophobicity increases, which leads to lower degradation and thus slower drug release rate (Engineer et al., 2011). Furthermore, the final Mw of the polymer also influences the degradation and drug release kinetics of the resulting formulations; i.e., with a decrease in the Mw, degradation as well as drug release rates both increase (Xu et al., 2017). Next, degradation, release kinetics, and the Mw also correlate with the size of the resulting NPs formulate. These are crucial factors for the therapeutic performance of PLGA NPs. Despite the higher drug loading potential of larger sized formulations, achieving a lower nano-size range is essentially important for the ability of the NPs to overcome biological barriers and to reach the disease site. In this context, a study pointed to the impact of the Mw of four 1:1 (LA:GA) PLGA copolymers with different Mw of 14.5, 45, 85, and 213 kDa on polymeric degradation and release rate (Mittal et al., 2007). With increasing Mw, the PLGA NPs degradation as well as its drug release decreased with a payload release under physiological conditions on day 18 of 95, 66, 50, and 23%, respectively. In addition it has been observed that the is higher the Mw of PLGA (6, 14.5, 63.6 kDa), the bigger is the size of NPs loaded with paclitaxel (PTX) (122 ± 3, 133 ± 2, 160 ± 2 nm) and also of NPs without PTX (117 ± 2, 132 ± 2, 159 ± 3 nm) (Fonseca et al., 2002; Song et al., 2008). LA/GA ratio is an effective parameter in tailoring degradation time and drug release rate. The higher the GA content, the faster the resulting degradation rate (Xu et al., 2017). *Vice versa* the drug release is prolonged with an increase in LA content (LA/GA: 50/50, 75/25 showed faster release kinetics then 100/0) (Horisawa et al., 2002). Hence, these polymeric characteristics, as well as their size, are important to tailor hydrophobicity, drug loading efficacy, and the pharmacokinetic profile of PLGA formulations.

Another important factor that affects the outcome of cancer treatment is the shape of PLGA NPs. Up to date, rod- (Bowerman et al., 2017), needle- (Kolhar et al., 2011), and cylindrical- (Chu et al., 2013) NPs have been synthesized and compared with the most frequently used spherical-shaped PLGA NPs regarding cellular uptake, internalization, biodistribution, and blood half-life. For instance, the needle-shaped PLGA NPs seem to more efficiently cross endothelial cell membranes, and deliver siRNA into the cellular cytoplasm (*in vitro*) as compared to their spherically shaped analogs. The mechanism of uptake is not yet fully understood but was narrowed down to three possible pathways: endocytosis, direct delivery and/or membrane portion with possible membrane disruption. The internalization was 150% higher with a threefold increased silencing efficacy compared to the spherical counterparts (Kolhar et al., 2011). However, the needle-shaped PLGA NP can also induce considerably cytotoxicity. After endocytosis the needle-shaped particles are in the lysosomes where they can damage the lysosomal membrane and by this activate apoptosis signaling pathways and finally induce cell apoptosis (Zhang et al., 2017).

Furthermore, cylindrical docetaxel (DTX)-loaded PLGA NPs accumulated less in liver, spleen, and lung in comparison with the free drug and DTX-loaded spherical NPs (Chu et al., 2013). Likewise, the shape of PLGA copolymer structure can influence the efficiency of drug encapsulation. Tao et al. (2013) demonstrated that star-shaped PLGA copolymer show higher DTX encapsulation efficiency (97%) compared to linear block copolymers (83%).

Next to the intrinsic properties of PLGA NPs, their surface modification plays an important role with respect to targeting strategy, biocompatibility, and blood half-life. The latter can particularly be increased when PLGA is combined with other polymers such as polyethylene glycol (PEG), polyvinyl alcohol (PVA), and d-α-tocopheryl PEG 1000 succinate (TPGS). Surface modification with, e.g., PEG (PEGylation) increases the hydrophilicity of the formulation yielding a stealth particle with enhanced blood circulation time and improved pharmacokinetics by preventing opsonization, and uptake by the mononuclear phagocyte system (Vllasaliu et al., 2014; Turecek et al., 2016). However, due to the same reasons, the cellular uptake by target cells (which might be mandatory depending on the treatment) might as well be decreased and the targeting capabilities of those particles are strongly dependent on the exact chemical composition of the surface. For instance, Khalil et al. synthesized curcumin (CUR)-loaded PLGA NPs with and without PEGylation via single emulsion solvent-evaporation technique and compared both formulations with the free drug, showing that PEGylation could improve the pharmacokinetic properties of the drug and the PLGA particle. The CUR biological half-life after per os administration was also increased for PEGylated PLGA NP formulations compared to non-PEGylated ones and the bioavailability of the loaded CUR could be increased by 55.4 times. However, despite improved pharmacokinetics, the stealth PEG-PLGA NP showed a slightly faster release of the drug as the PLGA NPs (Khalil et al., 2013). Another study by Bertrand et al. (2017) investigated, the impact of the PEG density on blood circulation times for various NPs sizes (55, 90, and 120 nm) with varying PEG density on the PLGA surface. Interestingly, results have shown that above a certain threshold of repeating units and Mw of the PEG chain (20 PEG chains (5 kDa) per 100 nm^2^) the circulation times of the PEG_5 kDa_-PLGA NPs were dependent on the PEG density rather than the size of the PLGA NP. This effect might be attributed to the shielding properties of the densed PEG surface and the resulting hydration of the PLGA NP. However, a further increase of the PEG density did not show an additional benefit, thus indicating that surface modification can solely alter the biodistribution to a certain extent. On the contrary, PEGylation can promote PLGA NPs degradation and consequently accelerates the loaded drug release. This phenomenon is attributed to the hydrophilic nature of PEG chains that absorb water and stimulate the decomposition of PLGA chains (Rafiei and Haddadi, 2017). Thus, PLGA properties can be further tuned by the introduction of “third party” components as shown for surface modification with stealth polymers. With respect to active targeting approaches, the attachment efficacy of targeting ligands to the NPs and their targeting specificity may vary. Besides the requirement of multiple production steps, their therapeutic effect is often less fruitful than expected. For instance, a previous study performed with prostate-specific membrane antigen (PSMA)-targeted PLA/PLGA-NPs containing DTX showed that the effect of active targeting was not as high as expected (Hrkach et al., 2012). This could be explained by, e.g., PEGylation. Backfilling of the targeted NP with hydrophilic polymers can cover and hide the targeting ligand, and by thus hindering it’s binding. Furthermore, incorporation of a targeting moiety might cause size enlargement in NPs and thus, may reduce its ability to cross biological barriers (such as the vascular wall and the extravascular stroma) and/or simultaneously increase the uptake by MPS (Lammers et al., 2012).

## PLGA-Based NPs Preparation Methods

**FIGURE 2:**
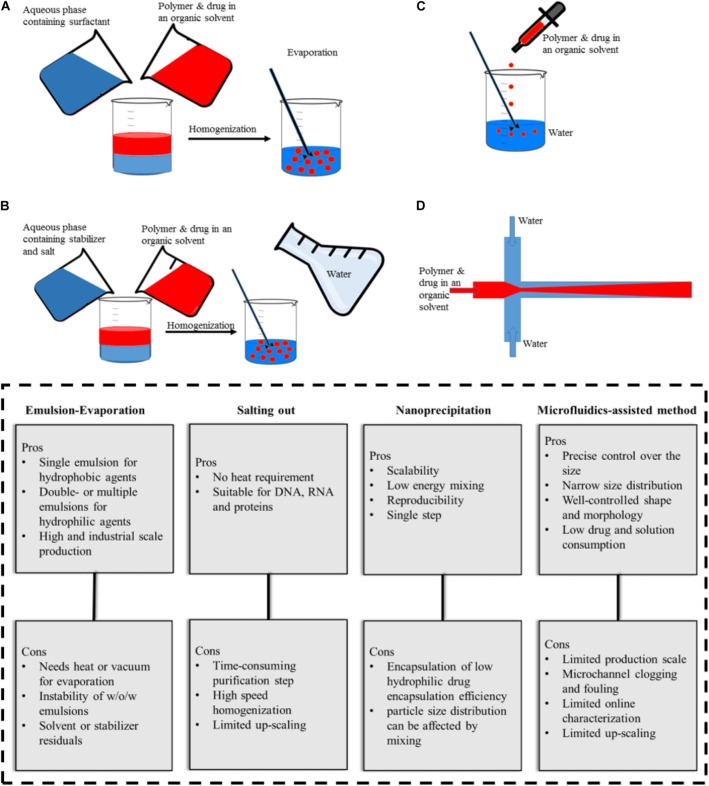
Illustration of PLGA NPs production methods and their advantages and disadvantages. **(A)** emulsification-evaporation, **(B)** salting-out, **(C)** nanoprecipitation, **(D)** microfluidic-assisted method.

The production techniques of NPs play an important role in their final features such as the shape, size, size distribution, and stability. A wide range of techniques have been used for PLGA-based NPs synthesis such as (single- or double-) emulsification, nanoprecipitation, dialysis, and spray drying. Herein, we highlight the most frequently used approaches (Figure 2) in the production of PLGA-based drug delivery nano-sized systems. For detailed information on rather sophisticated techniques, the reader can refer to previous publications (Sharma et al., 2016; Ding and Zhu, 2018).

### Emulsification-Evaporation Method

Emulsification is the most commonly used method for PLGA NPs production, where the drug dissolved in a volatile organic solvent is added to an aqueous phase containing surfactants under continuous stir. Subsequently, evaporation is applied to achieve the oil/water (O/W) emulsion form (Figure 2A). This procedure can be further followed by adding the resulting (O/W) emulsion to another aqueous solution to form a water/oil/water (W/O/W) (Kamaly et al., 2016; Masood, 2016). Alternative types of advanced emulsion techniques used for PLGA-based microparticle production based on W/O_1_/O_2_ or solid/oil/water (S/O/W) (Ramazani et al., 2016). For hydrophilic drugs the encapsulation efficiency is lower than in single emulsion compared to double- or multiple-emulsion techniques (Mendoza-Muñoz et al., 2016). The polymer concentration and evaporating step determine the NPs size. The higher the concentration of polymer in the discontinuous phase, the larger the size of the resulting particles. This method aims at the incorporation of a wide range of drugs, as well as, contrast agents (i.e., iron oxides) or the co-encapsulation of both substances in one formulation (Acharya and Sahoo, 2011; Lai et al., 2014; Mirakabad et al., 2014; Pérez et al., 2014; Shubhra et al., 2014). Despite the relatively poor loading of temozolomide in PLGA NPs, the single emulsion method performed best with respect to encapsulation efficiency compared with other preparation methods (Ananta et al., 2016). The pitfall of this commonly used method is the presence of surfactant residues on the NPs surface even after several washing steps (Pérez et al., 2014).

### Salting Out Method

In the salting-out method, a solution consists of polymer, drug, and a water-miscible organic solvent, which is added to an aqueous phase where salt and stabilizer are dissolved and stirred to form an emulsion (Figure 2B). The sudden introduction of water content causes the organic solvent to diffuse into the water and leads to NP formation (Mirakabad et al., 2014). The method is favorable for high concentrations of the polymer and also applicable to heat-sensitive drugs/agents since heat is not required during the process (Mir et al., 2017). However, it is not suitable for lipophilic drugs and its purification procedure is time-consuming since it requires several washing steps to remove stabilizers (Dinarvand et al., 2011).

Sengul et al. reported a method combining the salting out and emulsification approach to encapsulate meloxicam in PLGA NPs with varying Mw (PLGA (50:50 ratio), 5–15 and 40–75 kDa). In this context, the PLGA polymer with higher Mw produced the most stable NPs (Sengel-Turk et al., 2012).

### Nanoprecipitation Method

If a solution that consists of polymer, drug, and water-miscible organic solvent is added drop wise to an aqueous solution, the resulting precipitation process results in NPs (Figure 2C). This method is a straightforward single-step process with high reproducibility and was initially applied for hydrophobic drugs. Its advantages are scalability and low energy requirement where the NPs properties depend on polymer content and Mw, the nature of the solvents, and the ratios accompanied by mixing rate (Miladi et al., 2016). PLGA NPs (with various surface modifications such as PEGylation) and targeted PLGA NPs were prepared with this method and used to deliver anticancer drugs to the tumor site (Danhier et al., 2009; Valencia et al., 2012; Almoustafa et al., 2017). Further adjustments like pH variation and incorporation of salt additives and/or oil solutions have been applied to improve encapsulation efficiency (Rivas et al., 2017). For example, replacing water with cottonseed oil and Tween-80 as non-solvent made it possible to load hydrophilic drugs in PLGA NPs (Dalpiaz et al., 2009, 2016). In addition an increase in pH from 5.8 to 9.3 and exchanging procaine hydrochloride with procaine dehydrate resulted in an increase in drug entrapment from 11.0 to 58.2% (Govender et al., 1999). Niu et al. (2009) increased the encapsulation efficiency (>95%) of DNA into PLGA NPs with a modified nanoprecipitation method in which protein and polymer solution in DMSO nanoprecipitated in an aqueous solution of plaxomer. The authors also claimed that this method could outperform the conventional emulsion-evaporation techniques (encapsulation efficiency ∼65%). Moreover, Alshamsan (2014) showed that nanoprecipitation is more efficient than emulsion-based methods in encapsulating cucurbitacin I in PLGA.

### Microfluidics-Assisted Method

In microfluidic systems, small volumes (micro- or nano-liter) of liquids are processed in microchannels to achieve better results in comparison with the conventional bulk system. Depending on the type of reagents flow, microfluidic systems can be classified into two general types, (1) continuous-phase flow, and (2) segmented/droplet-phase flow microfluidic systems. The continuous phase flow microfluidics can be used to produce PLGA particles in the nanoscale while particles produced by segmented flow are typically in the micron size range. To produce PLGA nano-scale particles by continuous phase flow, an organic mixture of polymer and drug is assisted by a flow of aqueous phase solution on both sites along the microchannel, and the precipitation takes place in the organic phase (Figure 2D). PLGA NPs produced by microfluidic systems have several advantages; narrow size distribution, well-controlled NP synthesis by controlled reaction time and temperature, improved heat and mass transfer, expedited synthesis, and an overall high reproducibility rate from batch-to-batch (Valencia et al., 2011; Li and Jiang, 2018). Moreover, microfluidically produced NPs have a compact morphology, which can hinder initial burst release (Hasani-Sadrabadi et al., 2015). There are reports on microfluidic systems for the synthesis of various PLGA NPs (e.g., PEGylated, lipid coated and targeted NPs) (Valencia et al., 2013b) for anticancer drug loading ranging from simple two dimensional (Dashtimoghadam et al., 2016; Bramosanti et al., 2017), to more complex 3D (Kranz et al., 2000), and also origami designs (Sun et al., 2013).

## Drug Targeting

Typically, cancer therapies involve the systemic administration of drugs into the body or its oral uptake, both of which can damage healthy tissues by significant off-target accumulation and thus, generate serious side effects. Off-target accumulation limits the dosage that can be administered. To overcome this limitation, various targeting strategies are being investigated.

### Passive Targeting

Tumor accumulation of nanoformulations is first and foremost based on the enhanced permeability and retention (EPR) effect. A leaky, permeable tumor vasculature in combination with the lack of functional lymphatic drainage is a pathophysiological phenomenon, which leads to an enhanced accumulation of nanosized molecules in tumor tissue (Maeda et al., 2001). PLGA NPs characteristics such as high stability and tunable prolonged blood circulation time are ideal to use this so-called passive targeting approach (Danhier et al., 2012; Wicki et al., 2015).

**FIGURE 3:**
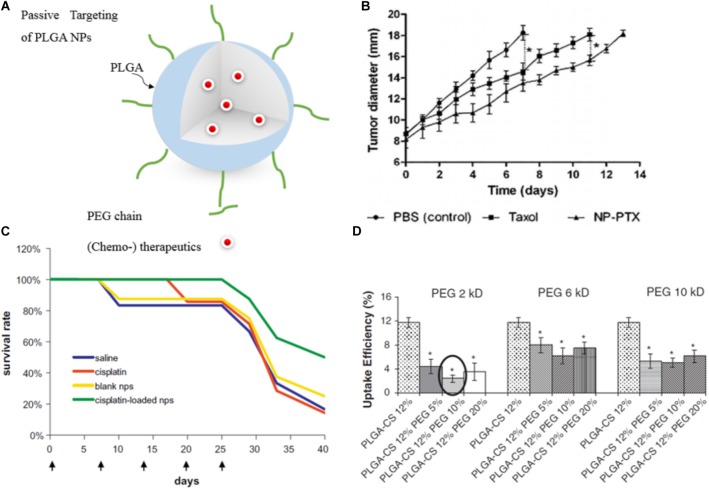
Examples of passively targeted drug-loaded PLGA NPs. **(A)** Illustration of a drug-loaded stealth PLGA NP. **(B)** Antitumor effect of passive targeted PTX-loaded NPs and PTX (Taxol^®^) on mice with orthotopic hepatocellular carcinomas. Untreated control animals received PBS injection. The control treatments groups received PTX-loaded NPs or PTX only. Highest response was shown for the nanomedicine formulation (^∗^*p* < 0.05). Reprinted from Danhier et al. (2009) with permission from Elsevier. **(C)** The survival rate of colorectal adenocarcinoma HT 29 tumor-bearing mice injected intravenously with cisplatin-loaded PEGylated-PLGA NPs is higher than for the free drug, naive control and vehicle control. Figure reprinted from Mattheolabakis et al. (2009) with permission from Elsevier. **(D)** Phagocytic uptake efficiency of PTX-loaded PLGA NPs in dependence of the surface modification with different amount of PEG with varying Mw. Results showed that NPs with 10% of 2 kDa PEG Mw, along with 12% CS were most efficient in terms of evading phagocytic uptake and consequently are expected to have the most prolonged blood circulation time (^∗^*p* < 0.05). Reprinted from Parveen and Sahoo (2011) with permission from Elsevier.

This passive targeting strategy has been applied for PLGA-based NPs encapsulating chemotherapeutics (Figure 3A) such as doxorubicin (DOX) (Park et al., 2009), PTX (Figure 3B) (Danhier et al., 2009), cisplatin (Figure 3C) (Mattheolabakis et al., 2009; Moreno et al., 2010), and CUR (Mohanty and Sahoo, 2010) to enhance antitumor activity, prolong circulation time and improve stability of the drug by protecting it from the blood components. For instance, CUR-loaded PEGylated PLGA nanocapsules with castor oil core exhibited increased blood circulation time to overcome CUR’s short half-life in the biological environment (Klippstein et al., 2015). Similarly, the elimination half-life of PEGylated PLGA-NPs loaded with DTX was shown to be 3.7-fold higher than observed for the free drug (Rafiei and Haddadi, 2017). For the passively targeted NPs, phagocytic uptake inversely reflects the blood circulation time since NPs with stealth properties can evade phagocytic uptake and thus circulate longer in the blood. Parveen and Sahoo evaluated PTX loaded PLGA NPs with various surface modifications [a blend of constant Chitosan (12%) and different PEG percentages (5, 10, and 20%) and Mws (2, 6, and 10 kDa)]. Results revealed that NPs with 10% of PEG (Mw = 2 kDa), along with 12% Chitosan were the most efficient NPs in terms of evading phagocytic uptake (Figure 3D) and consequently showed the highest blood circulation time (Parveen and Sahoo, 2011). Moreover, encapsulation of anticancer drugs within the biocompatible polymers and their passive delivery to the tumor site significantly reduced drug side effects. In line with this, also cisplatin encapsulated NPs induced no significant change in body weight and blood urea nitrogen (BUN) plasma levels in tumor-bearing mice (while the free drug resulted in an increase of both parameters) pointing to reduced side effects for the PLGA NPs compared to the free drug. Thus, the encapsulation of antitumor drugs into PLGA NPs not only improves antitumor efficacy but also can significantly reduce the side effects (Moreno et al., 2010).

However, passive targeting has several limitations (Lammers et al., 2012): the heterogeneous vascularization, and permeability, and the highly increased interstitial fluid pressure in many tumors are obstacles that limit the success of passive targeting. These, however, cannot be overcome just via NP adjustment. The EPR effect varies among patients, tumors, tumor types and even changes over time (Shi et al., 2017). This inter- and intra-tumoral variance of the EPR effect encouraged many researchers to question the existence and usefulness of this effect (Danhier, 2016). However, understanding the vascular tumor pathophysiology in more detail can help to address this limitation. For example, imaging protocols, which are able to visualize and quantify the extent of the EPR-mediated tumor targeting in individual patients, or histopathological biomarkers, can be used to predict nanomedicine accumulation and thus to select a responsive patient cohort (Lammers et al., 2012; Danhier and Préat, 2015). To facilitate the translation of NPs into the clinic, and to allow for individualized and improved anticancer nanomedicine therapies, several pharmacological and physical means can be employed to enhance the tumor accumulation and the efficacy of EPR-based nanomedicines. In the class of pharmacological strategies, the most prominent are treatments with drugs, which modulate vascular endothelial growth factor (VEGF) signaling, that act agonistic or antagonistic to angiotensin, enhance the local concentrations of nitric oxide, or, in case of tumor necrosis factor-α (TNF-α), enhance vascular leakiness. Other pharmacological strategies base on vessel promotion, with, e.g., recombinant human erythropoietin (Epo) or cilengitide, both inducing an increase in vessel density and in the relative blood volume and by this enhance drug delivery to the tumor site (Bridges and Harris, 2015; Doleschel et al., 2015; Wong et al., 2015; Nel et al., 2017). Alternatively, physical interventions can be applied including hyperthermia, radiotherapy and sonoporation. Hyperthermia typically leads to an increase in tumor perfusion and to an enhanced vascular permeability, thus promoting drug (and oxygen) supply to tumors (Kong et al., 2001). Radiotherapy can, next to an increase in vascular leakiness (Park et al., 2001), lead to a decrease in cell density within tumors and consequently also to a reduction in interstitial fluid pressure (Znati et al., 1996). In that line, microbubbles (which are routinely used as contrast agents for ultrasound imaging) can also be employed to temporarily increase vessel permeability and perfusion by sonoporation and thus increase drug delivery to the region of interest (Theek et al., 2014).

### Active Targeting

**FIGURE 4:**
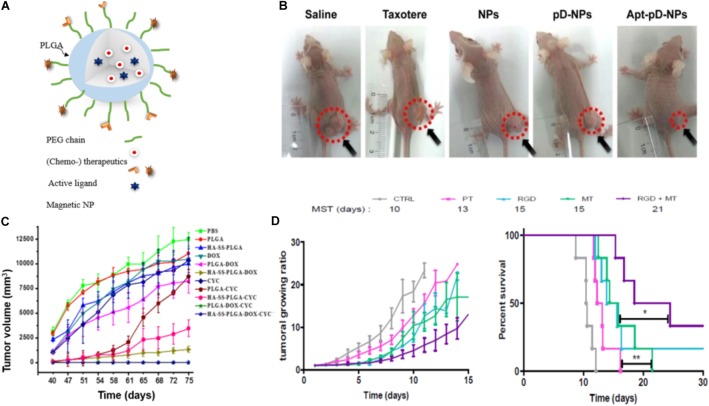
Examples of actively targeted drug-loaded PLGA NPs. **(A)** Scheme of a magnetically and molecularly targeted stealth PLGA NP. **(B)** Tumor growth inhibition was most effective for HeLa xenografted mice treated with DTX-loaded Aptamer-poly(dopamine)-NPs (Apt-pD-NPs) compared to all control groups (NPs: DTX-loaded PLGA NPs; pD-NPs: poly(dopamine) coated-NPs; Apt-pD-NPs: AS1411 aptamer conjugated pD-NPs). Image republished with permission of Dove Medical Press Ltd., from Xu et al. (2016). **(C)** Tumor volume curves after withdrawing of the different drugs at day 40. Breast cancer tumor-bearing mice were treated with PBS, PLGA NPs, hyaluronic acid-cystamine-PLGA (HA-SS-PLGA), free DOX, DOX-loaded PLGA NPs, DOX-loaded HA-SS-PLGA, free cyclopamine (CYC), CYC-loaded PLGA NPs, CYC-loaded HA-SS-PLGA, DOX- and CYC-loaded PLGA, DOX-and CYC-loaded HA-SS-PLGA NPs. Drug-loaded PLGA-HA particles (HA-SS-PLGA-DOX, HA-SS-PLGA-CYC and HA-SS-PLGA-DOX-CYC) and dual-drug-loaded particles (PLGA-DOX-CYC and HA-SS- PLGA-DOX-CYC) significantly delayed tumor recurrence, while tumors rapidly regrew in case of treatments without CYC. Reproduced from Hu et al. (2015) with permission of The Royal Society of Chemistry. **(D)** Tumor growth curves (left) and survival rate (right) of colon carcinoma bearing mice treated with either SPIONs/PTX co-loaded PLGA NPs (PT), RGD grafted on PT NPs (RGD), PT NPs with 4 h magnetic targeting (MT), and RGD grafted on PT NPs with 4 h magnetic targeting (RGD + MT). Tumor growth curves show no significant difference between the groups solely treated with active or magnetic targeting, while the combination of both (CityRGD + MT) increased the survival rate significantly (^∗^*p* < 0.05 and ^∗∗^*p* < 0.01). Tumor growth curves (left) and survival rate (right) reprinted from Schleich et al. (2014) with permission from Elsevier.

To increase the specificity of NPs for the target site and to promote cellular uptake, specific ligands can be attached to the surface of NPs (Figure 4A) that bind to receptors or antigens on tumor cells, the tumor microenvironment or the tumor vasculature (Perez-Herrero and Fernandez-Medarde, 2015).

Biotin, folic acid, aptamers, antibodies, and peptides are ligands that were used frequently in case of PLGA NPs (Table 1). For instance, DTX-loaded PLGA NPs surface modified with poly(dopamine) (a hydrophilic neutral polymer) and TPGS, functionalized with the DNA aptamer AS1411 improved tumor growth inhibition (Figure 4B) compared to all control groups, namely saline, Taxotere^®^, DTX-loaded NPs and most importantly passive targeted DTX-loaded poly(dopamine)-NPs. However, active targeting may also induce adverse effects: upon surface modification, circulation time is often decreased due to increased opsonization and recognition by the MPS (Theek et al., 2014). In addition, it has been stated that receptor expression on the tumor cells can change over time and next to pathway switching between different receptors, alternative receptors can be upregulated on the tumor cells (Zhu et al., 2018). These facts encouraged researchers to develop NPs that are targeting receptors that are overexpressed at different tumor cell differentiation states. For instance, hyaluronic acid functionalized NPs bind to the CD44 receptor, which is overexpressed in both breast cancer stem cells and regular cancer cells. These hyaluronic acid-cystamine-PLGA (HA-SS-PLGA) NPs that were loaded with DOX and an inhibitor of the hedgehog signaling pathway of cancer stem cell (cyclopamine) showed superior antitumor activity *in vitro* and *in vivo* (Figure 4C) due to internalization into both types of cells. Without combination treatment tumor cells proliferated after withdrawal of the drug in case of treatment with PLGA-NPs loaded with only one of each drugs (Hu et al., 2015).

**Table 1:** Examples of targeted PLGA NPs formulations: targeting ligands, anticancer drugs, polymer molecular weights and NPs sizes along with key results.

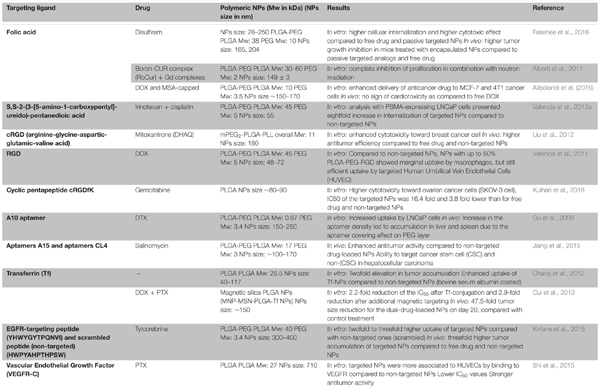

### Magnetic Targeting

Another (active) targeting strategy for drug delivery is magnetic guidance of NPs to the tumor site. In this approach, NPs are loaded with magnetic NPs, which are accumulated at the tumor site by applying an external magnetic field (Polyak and Friedman, 2009). For instance, Schleich et al. co-loaded SPIONs and the chemotherapeutic drug PTX into PLGA NPs and actively targeted them with RGD to αvβ3 integrins. NPs combining active and magnetic targeting promoted an up to eightfold increase in tumor accumulation compared to passive targeted NPs (no RGD or SPIONs), resulting in a prolonged survival rate of colon carcinoma (CT 26) xenografted mice and an inhibition in tumor growth (Figure 4D). While magnetic targeting improved the accumulation of the NPs in the tumor, penetration and internalization could not be promoted. This was mediated via the EPR effect and cellular uptake could be enhanced via conjugation of RGD mediating NPs’ binding to αvβ3 integrins on the tumor cells (Schleich et al., 2013, 2014).

In a similar approach, Al-Jamal et al. (2016) showed tumor growth inhibition in CT26 colon cancer tumor-bearing mice after magnetically targeted drug delivery of long circulating PEGylated-PLGA nanocapsules (NCs) co-loaded with DTX and varying amounts of SPIONs (0–7% w/w). They also reported that with increasing SPION load, the >200 nm sized NCs showed an increased uptake by liver and spleen, lower blood circulation time, and decreased tumor accumulation. Within the investigated period tumor uptake was significantly higher for tumors that were exposed to a magnetic field compared to tumors that were not exposed while the overall biodistribution of the NCs was not significantly altered by the magnetic field. All investigated NCs showed less systemic toxicity compared to the free drug. Moreover, they computed the magnetic, viscoelastic, convective and diffusive forces and extrapolated the observed efficiency from the rodent experiment to the human body (Al-Jamal et al., 2016).

Cui et al. (2016) evaluated a combination of chemotherapeutics (PTX and CUR) along with magnetic NPs encapsulated in PEGylated PLGA and active targeting with a transferrin receptor binding peptide. In line with the results reported previously, the authors reported that the combination of active and magnetic targeting increased the NPs accumulation and cellular uptake. In detail, a more than 10-fold higher tumor accumulation and improved crossing of the blood–brain barrier by fivefold was achieved in an orthotopic glioma model compared with non-targeted NPs. The application of the magnetic field in combination with transferrin receptor targeting also resulted in an increase in the overall survival rate of the animals and a higher therapeutic efficacy as compared to control groups treated with either magnetic or active targeting alone. However, indications for magnetic targeting are limited by of the difficulty to focus the magnetic field to deep body locations. One loophole for that limitation can be the application of magnetic particle imaging (MPI). Iron oxide NPs are responsive to a given magnetic field and the magnetic moments will line up in the direction of the induced magnetic field. In this context MPI might not only be used as a non-invasive imaging modality, but also promote magnetic targeting and guide the, e.g., SPIONs to the target tissue. Additionally, MPI can be combined with magnetic hyperthermia as the SPIONs can be excited to generate heat yielding an image-guided theranostic tool (Bauer et al., 2016) (see section “Magnetic Hyperthermia”). Nevertheless maintaining the high magnetic gradient strength needed for clinical translation remains challenging and costly (Tay et al., 2018).

## Combination Treatments With PLGA NPs

### Clinically Approved PLGA NPs—Lost in Translation?

Chemotherapeutic PLGA formulations with varying properties (i.e., shape, carrier, size, etc.) are currently available and FDA-approved for several types of cancer treatments. The most prominent among them are based on PLGA microspheres, namely Lupron Depot^®^ (Abbvie Endocrine Inc.) and Trelstar^®^ (Allergan Sales Inc.). Other PLGA formulations in the market are PLGA-based gels (e.g., Eligard^®^, Tomar Therap) and implants (e.g., Zoladex^®^, Tersera Theraps LLC.). In a set of PLGA NPs with varying LA/GA ratios tested, the only NP formulation, which was further used in clinical trials did not contain GA and the NP was synthesized from PLA-PEG. This first targeted PLA NP (BIND-014, BIND Therapeutics) encapsulating DTX was applied for targeting the PSMA and the neovasculature that over-expresses PSMA receptor (Von Hoff et al., 2016). The early clinical trials of BIND-014 reported an enhanced anti-tumor activity compared with conventional DTX. Reasonably, many further potential clinical applications of this drug delivery system family for various cancer types, e.g., urothelial carcinoma, metastatic prostate cancer and squamous cell carcinoma of the head and neck were mentioned in related clinical trials (NCT02479178, 2016). However, the study failed in phase II due to low response rates and the company was sold with substantially all of BIND’s assets to Pfizer in 2016.

With respect to drug development, researchers often need to take a step back to the fundamental research to continue improving anticancer drug delivery such as enhancing hydrophilicity, reducing uptake by the MPS, increase tumor to background ratios and tumor-targeted specificity to achieve a higher response rate (Chidambaram et al., 2011).

In daily clinical practice patients often receive a combination treatment like, e.g., AC- or TAC-chemotherapy (A = Adriamycin (DOX); C = cyclophosphamide; T = Taxotere (DTX)) for breast cancer or BEAM-chemotherapy (BICNU^®^, Etoposide, Ara-C cytarabine and melphalan) for the treatment of lymphomas.

The synergism of those standard combination treatments might be increased by the use of nanoformulations and researchers are evaluating their potential *in vitro* and in pre-clinical *in vivo* studies. The NPs offer a platform to co-encapsulate various pairs of drugs with varying hydrophobicity and pharmacokinetic profiles ensuring simultaneous long term distribution to the target site. Such NP co-formulations can extend the drugs’ circulation time (Tian et al., 2017), sustain drug release (Khuroo et al., 2018) and also inhibit development of drug resistance (Misra and Sahoo, 2011) as well as increase drug accumulation in tumors (Afrooz et al., 2017). Among the many combinations co-loaded in one particle are PTX-Cisplatin (Tian et al., 2017), PTX-Erlotinib (Khuroo et al., 2018), DOX-CUR (Misra and Sahoo, 2011), PTX-Verapamil (Afrooz et al., 2017), and PTX-epigallocatechin gallate (EGCG). The latter was used for the sequential release of first EGCG and second PTX from PLGA-based core-shell NPs to improve therapeutic efficiency. In this context, the multiple signaling inhibitor EGCG was released first and increased the sensitivity of resistant breast cancer cells toward PTX as indicated by the suppression of the permeability glycoprotein (Pgp) (Narayanan et al., 2014, 2015).

Next to the synergistic effect of different chemotherapeutic drugs, an additional beneficial therapeutic outcome can be achieved via combination treatment with radiotherapy. Ionizing irradiation can increase vascular leakiness and furthermore lead to a decrease in cell density within tumors, and thus reduce the interstitial fluid pressure (Znati et al., 1996; Peschke et al., 1999). These effects contribute to an increased accumulation of both low-molecular-weight drugs and nanomedicine formulations in tumors (Higgins et al., 2015). Consequently, the efficacy of nanomedicine-based chemotherapy can increase (Wasan et al., 2017). *Vice versa* radiosensitizers can be used to improve the effect of radiotherapy (Jin et al., 2007; Wang et al., 2010; Kwatra et al., 2013; Bergs et al., 2015). The most prominent radiosensitizing drugs are PTX, DOX, and DTX. In this context, Werner et al. encapsulated DTX in PLGA NPs with a lecithin-PEG folate surface modification to optimize radiotherapy. Results revealed that radiation 12 h post NPs administration had a maximum effect on tumor growth inhibition (Werner et al., 2011). In a comparable study, prostate cancer cell penetrating peptide (R11) conjugated PLGA NPs were used for targeted radiosensitization of prostate cancer cells leading to higher antitumor activity under the exposure (Menon et al., 2015).

### Magnetic Hyperthermia

**FIGURE 5:**
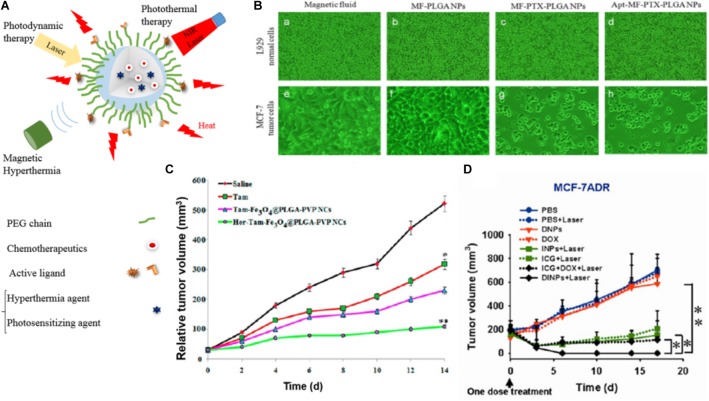
Examples of the performance of PLGA-based NPs used for magnetic hyperthermia, photodynamic therapy, and photothermal therapy drug-loaded PLGA NPs. **(A)** Illustration of drug-loaded NPs used for magnetic hyperthermia, photodynamic therapy, and photothermal therapy. **(B)** Microscopic images of fibroblasts (L929) and MCF-7 cells 5 days after treatment with MF, MF-PLGA, MF-PTX-PLGA, and Apt-MF-PTX-PLGA NPs indicate that actively targeted NPs have the highest cytotoxicity toward cancer cells *in vitro*. Images reprinted from Aravind et al. (2013) with permission from Elsevier. **(C)** Tumor volume changes in breast cancer xenografts in mice treated with PLGA NPs co-loaded with tamoxifen (TAM) and Fe_3_O_4_ for hyperthermia with and without trastuzumab. Reprinted with permission from Vivek et al. (2016). Copyright (2016) American Chemical Society. **(D)** Tumor growth curves of different groups of mice inoculated with drug resistant breast cancer cells (MCF-7/ADR) after treatments with DOX and ICG co-loaded PLGA@lecithin@PEG NPs (DINPs) prove the added value of PDT (^∗^*p* < 0.05 and ^∗∗^*p* < 0.01). Figure reprinted with permission from Zheng et al. (2013). Copyright (2013) American Chemical Society.

In hyperthermia the body temperature is increased to damage and kill tumor cells. Hyperthermia can be applied to the whole-body or regionally. The techniques used to induce local hyperthermia are radiofrequency, (high intensity focused) ultrasound, microwave, and laser irradiation as well as alternating magnetic fields. Regarding the latter, PLGA is considered as a suitable carrier to load and deliver magnetic NPs and (chemo-) therapeutics (Figure 5A). It has also been indicated that PLGA encapsulation can improve SPIONs stability without changing or affecting the photothermal ability of the nanocomposites (Sivakumar et al., 2017). For local hyperthermia SPIONs or fluid containing magnetic NPs have been injected into the tumors and an alternating magnetic field applied to raise the temperature to 42–46°C. The efficiency of magnetic hyperthermia depends on the size and magnetization of the particles and also on the maximum temperature that can be reached by the particle (Curie temperature) (Thorat et al., 2016).

Hyperthermia is generally applied in combination with chemotherapy or radiotherapy (Rao et al., 2010; Cabuy, 2011; Kaur et al., 2011). Eynali et al. (2017) encapsulated 5-FU into 30–100 nm PLGA NPs with and without an iron oxide core and showed a reduction in the proliferation rate of the colon cancer cell line HT-29 for the combination treatment of hyperthermia and chemotherapy *in vitro*.

In a similar setup Aravind et al. designed and evaluated a multimodal theranostic PLGA nanocomposite containing a magnetic fluid of Fe_3_O_4_ for hyperthermia induction and MRI imaging, the chemotherapeutic drug PTX, and the fluorescent dye Nile red (NR). Additionally the surface was conjugated with an aptamer targeting the nucleoline receptor yielding a particle with an overall size of 218 nm (Aravind et al., 2013). The *in vitro* analysis showed that the aptamer ligand on the NPs surface increased the tumor cell (MCF7) uptake while lowering the uptake in normal, mouse fibroblast cell line (L929) and thus lowered the *in vitro* cytotoxicity at 5 days after treatment (Figure 5B).

In agreement with these findings Vivek et al. evaluated a multifunctional pH-sensitive nanocarrier (PLGA-PVP (polyvinylpyrrolidone) NPs) co-loaded with tamoxifen (TAM) for chemotherapy and Fe_3_O_4_ for hyperthermia with and without Herceptin on the surface for targeting the human epidermal growth factor receptor 2 (HER 2). They observed a 90% higher reduction in tumor sizes for actively compared to passively targeted NPs (60% reduction) in mice 14 days post-injection (Figure 5C). In addition, immunohistochemistry did not reveal any visible morphological change of healthy tissue after treatment with the PLGA-PVP (polyvinylpyrrolidone) NPs (Vivek et al., 2016). However, the superiority of actively targeted versus passively targeted nanocomposites could as well be an effect of the additional therapeutic interaction of the Herceptin, which upon binding to HER2 prevents the cells from proliferating.

In general, promising therapeutic efficacy could exclusively be achieved via combination treatments; neither the sole delivery of chemotherapy nor the local magnetic hyperthermia could outperform the proposed and presented combination treatments. This can be due to synergistic effects and/or due to an increased drug accumulation following the hyperthermia-related enhancement of the EPR effect. However, despite those promising pre-clinical results (and as already discussed for magnetic targeting) the challenges for successful clinical translation remain unchanged. Importantly, even with MPI-guided induction of hyperthermia, the main challenge is to meet the clinical SPION dose limits and still achieve a therapeutically relevant heating in the region of interest (Tay et al., 2018).

### Photodynamic and Photothermal Therapy

In photodynamic treatments a photosensitizing agent (PS) is applied to generate highly ROS via photoexcitation (Chatterjee et al., 2008; Paszko et al., 2011; Calixto et al., 2016). However, most of the PS agents are hydrophobic, show a rapid decomposition under laser irradiation, do not accumulate well in the tumor and cannot be efficiently excited in the near infrared range (which is prerequisite for reaching deeper tissues). Nanocarriers like PLGA-NPs can be used to overcome some of these drawbacks (Paszko et al., 2011; Lim et al., 2013). In line with this, the most commonly used PS agents that have been loaded into nanocarriers like PLGA NPs are poly(anilin) (Nguyen et al., 2017), indocyanine green (ICG) (Zheng et al., 2013; Lee and Chang, 2017), and zinc(II)-meso-tetraphenylporphyrin (ZnTPP) (Boix-Garriga et al., 2015).

Among them, ICG is not only a suitable PS agent, but is also categorized as a photothermal agent and in addition allows for optical imaging. In this context, Zheng et al. designed a lipid-polymer hybrid NP (PLGA-lecithin-PEG) that was co-loaded with DOX and ICG for combinational chemo- and photodynamic treatment. The NPs were synthesized by a single-step sonication method with an average diameter of 90 nm and were evaluated *in vitro* as well as *in vivo* in chemotherapy resistant (MCF-7/ADR) and sensitive (MCF-7) breast cancer cells. After laser irradiation, the combined treatment of DOX and ICG-loaded PLGA-lecithin-PEG synergistically promoted cell death and tumor growth inhibition in both tumor models (Figure 5D) (Zheng et al., 2013; Lee and Chang, 2017).

In photothermal therapy (PTT) a therapeutic agent absorbs photon energy and dissipates it in form of heat. This heat results in the disruption of cellular membranes and induces apoptosis and/or necrosis. PTT can be applied with various organic nanomaterials (e.g., carbon and gold nanomaterials, etc.) and polymeric NPs with NIR-absorbing agents like ICG (Cheng et al., 2014). In that line PLGA and its copolymers (e.g., PLGA-PEG, PLGA-lipid) have been used for co-loading and combination therapy with, e.g., DTX and gold (Au) (Hao et al., 2015), DTX and poly(dopamine) (Peng et al., 2018) or DTX and polypyrrole (PPy) (Yuan et al., 2016). The latter PTT agent, PPy, is considered an ideal heat inductive material, which was used by Yuan et al. to build a DOX encapsulated PLGA-PPy NP. An additional surface modification with 2-Deoxy-glucose terminated PEG resulted in a sugar promoted 17-fold increased uptake of the NPs by MCF-7 cells without altering the photothermal properties of the compound. In a comparable NP design Peng et al. synthesized PLGA-NPs with a poly(dopamine) (PDA) coating modified with TPGS, yielding PLGA NPs@PDA-TPGS. Those NPs were loaded with DOX, and the combination treatment was evaluated *in vitro* and *in vivo* in BALB/c MCF-7 (drug resistant) breast cancer xenografted mice. The NIR irradiation triggered DTX release, and a synergistic antitumor efficacy indicated that the DOX-loaded PLGA NPs@PDA-TPGS could overcome drug resistance.

Next to the therapeutic effect of the generated heat and/or triggered drug release, PTT can like (magnetic) hyperthermia increase vascular permeability and blood flow and thus enhance EPR effect and increase drug delivery (Chen et al., 2006; Yuan et al., 2016).

Even though the combination treatment of chemotherapy and PTT/PDT holds great potential, also with respect to the synergistic effect on therapeutic outcome, the sites for PDT are limited by the penetration depth of the applied laser light (max. 1–2 cm), as well as by the short migration distance of the produced oxygen radicals (Moan and Berg, 1991). For optimal efficacy, photodynamic therapy, therefore, has to be directed to specific (sub-) cellular targets (mitochondria, lysosomes, or the cell membrane), which can be challenging in a clinical setting.

### Gene Therapy

Next to standard chemotherapeutic treatments and immunotherapies, gene delivery and gene silencing are emerging approaches in the anticancer field. Here, a double-stranded DNA (dsDNA) or a single-stranded DNA (ssDNA) is used for replacing (or completing) a gene while short interfering RNA (siRNA) is typically used for silencing a gene (Ibraheem et al., 2014). When cancer is initiated by cellular mutation, siRNAs can inhibit genes responsible for multidrug-resistance and in combination with, e.g., targeted chemotherapeutics, the self-defense mechanism can be inhibited (Xiao et al., 2017). The major obstacle in gene therapy is the delivery of the large, negatively charged and very fragile nucleic acids into the cell cytoplasm, respectively, the cell nucleus. PLGA has been used as a carrier to protect and deliver nucleic acids (Wang et al., 2016). Tang et al. used calcium phosphate–pDNA complexes embedded in PLGA NPs, which were able to inhibit the proliferation of breast cancer cells (4T1) and induce apoptosis. The transfection of the cells with the plasmid DNA encoding for the MsurvivinT34A gene resulted in tumor angiogenesis inhibition and a decrease in microvessel density. Furthermore, in combination with Doxil (DOX encapsulating liposomes) a significant suppression of tumor growth could be observed (Tang et al., 2014).

Recently, the PLGA nanomedicine platform has also been used for genome editing via the CRISPR (Clustered Regularly Interspaced Short Palindromic Repeats) technology. Delivery of the CRISPR-associated protein-9 nuclease (Cas9) complexed with a synthetic guide RNA (gRNA) into a cell enables genome cutting at a desired location, allowing existing genes to be silenced, removed or added. In chronic myeloid leukemia (CML) reciprocal translocation of chromosome 9 and 22t results in a breakpoint cluster region-abelson (BCR-ABL) fusion oncogene, which translates a relating BCR-ABL fusion protein. In a study by Liu and colleagues, this BCR-ABL fusion protein was knocked out via the delivery of a CRISPR/Cas9 plasmid (pCas9/gBCR-ABL), which was encapsulated in a PEG-PLGA-based cationic lipid-assisted polymeric NP. Successful knockout of the gene of interest was confirmed *in vitro* as well as *in vivo* in a CML mouse model. Mice that were i.v. injected with the nanocarrier showed a reduced number of myelogenous leukemia cells (K562) in blood and bone marrow and a significant prolonged survival rate after the treatment compared to naive and vehicle controls (Liu et al., 2018).

Challenges remain regarding the simultaneous entrapment and encapsulation of gene- and chemotherapy agents in polymeric NPs (Lee et al., 2016; Wang et al., 2017). In this context, pioneering work was done by Yang et al. who synthesized a DOX encapsulated PLGA-NP with a surface modification by a positively charged poly(ethyleneimine) for adsorption of condensed shRNA onto the NP, yielding a theranostic nanobubble that could also be visualized by ultrasound imaging. The shRNA downregulated the P-glycoprotein associated with the adenosine triphosphate-dependent drug efflux pump. This P-glycoprotein knockdown accelerated cellular uptake of the NPs, and due to the suppressed drug efflux increased the nuclear accumulation of DOX in drug resistant tumors (MCF-7/ADR) (Yang et al., 2015). In another approach, Zhang et al. used PEG-PLGA-poly (L-lysine) NPs to co-delivery DOX with siRNA. The presented NPs were surface modified with epidermal growth factor (EGF) for targeted delivery and treatment of lung cancer. The siRNA knocks down the anti-apoptosis protein B-cell lymphoma 2 and thus induced apoptosis in the targeted tissue. Biodistribution and therapy studies were performed using xenografts of the human non-small cell lung carcinoma cell line (H1299) in mice. The combination treatment suppressed lung cancer growth, and reduced expression of the anti-apoptosis protein, which was confirmed by *ex vivo* analysis of the tumor tissue (Yang et al., 2015; Yin et al., 2015; Zhang et al., 2016).

### Cancer Immunotherapy

Cancer immunotherapy can be performed via cancer vaccines, cytokine therapy, checkpoint-blockade therapy, adoptive T-cell transfer, and chimeric antigen receptor T (CAR-T) cell therapy (Yoon et al., 2018). Here, PLGA-based NPs can not only protect the sensitive cargos (e.g., antigens, adjuvants, etc.) from degradation but can also promote passive accumulation by the EPR effect and can facilitate additional active targeting strategies via surface modification. However, the stimulation of the immune system, which enables the recognition and attack of malignant cells, does not solely rely on tumor accumulation, but might as well be achieved or enhanced by targeting of immune cells, e.g., in liver, spleen, and lymph nodes. Thus, high affinity of many NPs to cells of the mononuclear phagocyte system as well as macrophage uptake might even be used to promote the desired immune-response (Jiang et al., 2017a,b).

In that regard, NPs can be used to deliver, e.g., immunogenic cell death (ICD) promoters, vaccines or immune checkpoint inhibitors through intravenous injection. Zhao et al. (2016) showed that mPEG-PLGA based NPs loaded with the chemotherapeutic ICD promoter oxaliplatin lead to a high response in an *in vivo* mouse model of pancreatic cancer. As a non-immunogenic control the same PLGA-NPs were loaded with the chemotherapeutic gemcitabine leading to a less effective therapeutic response (Zhao et al., 2016).

In another study, PLGA NPs coated with an agonistic αCD40-monoclonal antibody (mAb) were applied for vaccine delivery to dendritic cells (DCs) as the main antigen presenting cells. The PLGA NPs were loaded with the adjuvants Pam3Csk4 (a synthetic triacylated lipopeptide to stimulate toll-like receptor 2) and Poly (I:C) (Polyinosinic:polycytidylic; an immune-stimulator to toll-like receptor 3) to induce potent CD8^+^ T cell response. *In vivo* experiments in murine melanoma-OVA mouse model indicated that active targeting of DCs and vaccine delivery resulted in efficient priming of CD8^+^ T cells, tumor control, and prolonged survival of the tumor-bearing mice (Rosalia et al., 2015).

Furthermore, the use of cells as vaccines for generation of effective and adaptive immune responses is well established in cancer immunotherapy. Ahmed et al. coupled γ-irradiated non-vital prostate cancer cells with PLGA NPs. The particle-cell hybrid was then additionally functionalized with the adjuvant CpG ODN, which selectively activates the toll-like receptor 9. This activation resulted in efficient cancer vaccination in a prostate cancer model. Interestingly, comparable results could not be achieved with a similar particle-melanoma cell hybrid. Due to this, the authors concluded that a careful selection of the tumor entity in such hybrid NPs might be a prerequisite to elicit DCs (Ahmed et al., 2017).

Moreover, a combination of cancer immunotherapy with other modalities like PTT can provide a more effective treatment (Chen et al., 2016). In this context, multifunctional PLGA-PEG NPs co-loaded with ICG (photothermal and PS agent) and imiquimod (R837), an immune-adjuvant TLR-7 agonist, triggered vaccine-like immune responses. Additional combination of this NIR heater-loaded PLGA-based nanovaccine with an anti-cytotoxic T-lymphocyte antigen-4 checkpoint-blockade therapy synergistically inhibited the growth of metastasis and prevented recurrence of cancer.

## Conclusion

In this review article, we summarize the physicochemical properties of polymers and NPs based on PLGA, and discuss their preparation methods, and applications for various drug delivery approaches. While several different PLGA-based therapeutics are already routinely used in the clinic, despite promising pre-clinical results, PLGA-NPs are neither listed nor approved and the only formulation (BIND-014) that made it into clinical trials was based on PLA-PEG and failed in phase II due to non-responders.

**FIGURE 6:**
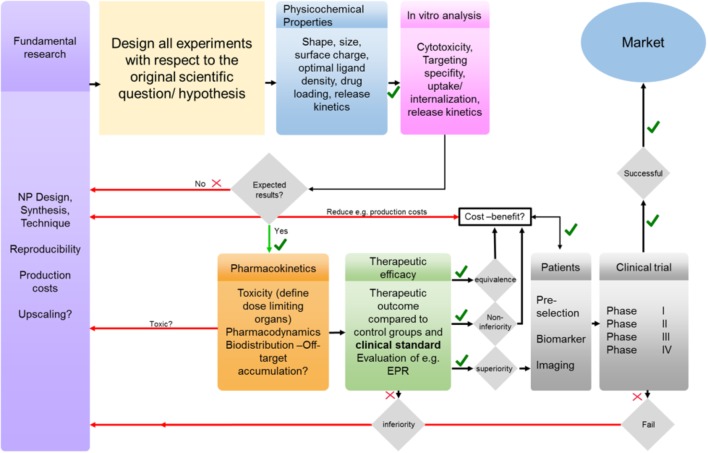
From bench to bedside: simplified illustration of several barriers and challenges that need to be tackled to achieve clinical translation. In fundamental research NP design and production should be performed with respect to a clinical relevant scientific question and all experiments should be designed accordingly and not *vice versa*. The effect of NPs physicochemical properties on their *in vivo* performance needs to be evaluated and optimized if necessary. Any failures in *in vitro*, *in vivo* and/or clinical trials need to be addressed and reconsidered in fundamental research.

As a consequence, researchers need to critically reflect the clinical feasibility of their approaches and develop NPs that better match the biomedical need (Figure 6). This not only relates to the optimization of size, drug loading and drug release, but also to biocompatibility, pharmaceutical upscaling and batch-to-batch reproducibility. In this context, it is advantageous if the experimental setup is from beginning on tailored to a concrete clinical problem. Especially in the case of PLGA NP synthesis batch-to-batch consistency (due to the heterogenous mixing conditions required for self-assembly), which directly relates to drug loading efficacy, and the challenge to obtain particle sizes below 100 nm in an up-scalable process are remaining issues regarding PLGA-NP production. The lack in reproducibility and the need for additional purification steps impede manufacturing according to good manufacturing practices (GMP) that is mandatory for successful clinical translation. Furthermore, awareness of pharmacokinetics and pharmacodynamics of the NPs is mandatory to avoid off-target accumulation and potential side effects. The (often too large) sizes of NPs as well as their surface charge can result in severe off-target accumulation and can even prevent targeted NPs from binding to the target tissue/cell. Surface modifications, yielding, e.g., in a stealth NP, might avoid the recognition by the immune system but will simultaneously increase the size, may prevent penetration of the nanomedicine, hide targeting motifs, and reduce cellular uptake (which is mandatory for the therapeutic effect of many drugs). Any even minor chemical modification of a nanomedicine formulation can alter the physicochemical properties and thus the *in vitro* and *in vivo* performance as well as therapeutic efficacy. Furthermore, when combining nanomedicines with approaches like PDT/PTT or magnetic guidance, one needs to consider that these concepts of drug delivery will only be operant if the external stimulus (e.g., magnet) can be applied to the target region of the human body (which is often not the case for tumors located deeply below the skin).

Next to the influence of the NPs design and the challenges regarding the application of (external) stimuli-induced delivery and cancer treatment, it has been increasingly recognized that the high inter- and intra-individual heterogeneity of the EPR effect is an important reason for the moderate clinical translation of many nanomedicine formulations including PLGA-NPs. Thus, nanomedicine research will strongly take advantage from concepts that consider and address pathophysiological features that impact the EPR effect and affect the accumulation, penetration, distribution, retention and efficacy of NPs formulations.

In conclusion, for an efficient clinical translation a more rational design of PLGA-NPs, along with clinically relevant preclinical therapy settings, in which nanomedicines are tested are mandatory to close the currently huge gap between material research, preclinical experimentation and clinical reality.

## Author Contributions

FK contributed toward conceptualization and planning of the manuscript. SR and ND wrote the paper and designed the figures. SR, ND, NG, and TL contributed toward data collection. FK, MM, TL, EK, YS, and FK contributed toward revising the paper and agree to be accountable for all aspects of the work. All authors agreed on the finally submitted version of the manuscript.

## Conflict of Interest Statement

The authors declare that the research was conducted in the absence of any commercial or financial relationships that could be construed as a potential conflict of interest.
